# Feasibility of one breath-hold cardiovascular magnetic resonance compressed sensing cine for left ventricular strain analysis

**DOI:** 10.3389/fcvm.2022.903203

**Published:** 2022-08-12

**Authors:** Xiaorong Chen, Jiangfeng Pan, Yi Hu, Hongjie Hu, Yonghao Pan

**Affiliations:** ^1^Affiliated Jinhua Hospital, Zhejiang University School of Medicine, Jinhua, China; ^2^Sir Run Run Shaw Hospital, Hangzhou, China

**Keywords:** compressed sensing, cardiovascular magnetic resonance, myocardial strain, ventricular function, agreement assessment

## Abstract

**Objective:**

To investigate the feasibility of 3D left ventricular global and regional strain by using one breath-hold (BH) compressed sensing cine (CSC) protocol and determine the agreement between CSC and conventional cine (CC) protocols.

**Methods:**

A total of 30 volunteers were enrolled in this study. Cardiovascular magnetic resonance (CMR) images were acquired using a 1.436 T magnetic resonance imaging (MRI) system. The CSC protocols included one BH CSC and the shortest BH CSC protocols with different parameters and were only performed in short-axis (SA) view following CC protocols. Left ventricular (LV) end-diastole volume (EDV), end-systole volume (ESV), stroke volume (SV), and ejection fraction (EF) global and regional strain were calculated by CC, one BH CSC, and shortest BH CSC protocols. The intraclass correlation coefficient (ICC) and coefficient of variance (CV) of these parameters were used to determine the agreement between different acquisitions.

**Results:**

The agreement of all volumetric variables and EF between the CC protocol and one BH CSC protocol was excellent (ICC > 0.9). EDV, ESV, and SV between CC and shortest BH CSC protocols also had a remarkable coherence (ICC > 0.9). The agreement of 3D LV global strain assessment between CC protocol and one BH CSC protocol was good (ICC > 0.8). Most CVs of variables were also good (CV < 15%). ICCs of all variables were lower than 0.8. CVs of all parameters were higher than 15% except global longitudinal strain (GLS) between CC and shortest BH CSC protocols. The agreement of regional strain between CC and BH CSC protocols was heterogeneous (-0.2 < ICC < 0.7). Many variables of CVs were poor.

**Conclusion:**

Notably, one BH CSC protocol can be used for 3D global strain analysis, along with a good correlation with the CC protocol. The regional strain should continue to be computed by the CC protocol due to poor agreement and a remarkable variation between the protocols. The shortest BH CSC protocol was insufficient to replace the CC protocol for 3D global and regional strain.

## Introduction

Assessing myocardial function is one of the most important steps in the management of cardiovascular diseases. Left ventricular (LV) ejection fraction (EF) is the key parameter for assessing clinical, normal, mildly reduced, or reduced cardiac function that can be confirmed by different EF ranges (1). Echocardiography, contrast-enhanced cardiac computed tomography, cardiac magnetic resonance (CMR), and single-photon emission computed tomography can be used to obtain the EF value. Among these techniques, CMR is considered as the gold standard method to obtain the EF value owing to its excellent tissue resolution, good spatial and temporal resolution, large field of view, and remarkable inter- and intra-observer agreement (1). CMR short-axis (SA) cine has been recommended and is routinely used for EF calculation. CMR is time-consuming and requires multiple breath-holds (BHs), which lower patients’ compliance and induce involuntary motions. Long scan times can also increase the probability of premature occurrence in patients with arrhythmia, resulting in motion artifacts and space blurring (2).

Myocardial strain is an emerging concept that represents deformation of the myocardium, which is also a parameter for myocardial motion (3). Recent studies (4–6) have reported that feature tracking is reproducible and reliable for strain analysis. The myocardial strain was considered superior in EF-preserved patients and played an important role in patients with subclinical LV dysfunction (7). SA along with long-axis view cines can be used to compute 3D global and regional strains, including radial, circumferential, and longitudinal. SA cine is important for circumferential strain and 3D strain analyses.

Multiple BHs and a long scan time are necessary for both EF and strain analyses. Reduced BHs and scan time would improve patients’ compliance, and accelerated SA cine acquisition can be an acceptable approach. Accelerated CMR cine techniques [such as k-t blast, parallel imaging, and compressed sensing (CS)] have been used in the quantitative assessment of ventricular volume and function (8–10). Both scan time and BHs were reduced drastically when parallel imaging or CS techniques were used.

Compressed sensing has been used to accelerate CMR imaging under the assumption that the k-space data are randomly under-sampled. A non-linear reconstruction was performed to enforce the sparsity of the image and consistency with the acquired magnetic resonance imaging (MRI) data (11). Recent studies (10–12) have reported that the CS techniques have been used in accelerated CMR acquisition, including 3D whole heart late gadolinium enhancement, whole heart contrast-enhanced magnetic resonance angiography, and SA cine. The studies (13–16) on CS cine (CSC) have reported that SA cine can be acquired in one BH without compromising image quality. The EF calculated by CSC acquisition is well correlated with the EF computed using conventional multiple BH cines. CSC significantly reduced the scan time, indicating that it can potentially replace conventional cine (CC) in EF analysis. Recent studies (10, 17) have reported no significant difference in global circumferential strain (GCS) and the GCS rate between one BH CSC and CC protocols, and excellent agreement has been reported between both types of cines. These findings initially indicated that accelerated CS cine CMR can be used in strain analysis. However, the importance of global radial strain (GRS) and global longitudinal strain (GLS) by using the CSC protocol remains unknown. Three-dimensional strain with SA and long-axis cines is considered superior to 2D strain using SA cine for global strain analysis, which could provide GCS, GLS, and GRS. The reliability of 3D global and regional strains by CSC is unknown to date. In the United Imaging MRI system, united compressed sensing (uCS) comprehensively combines all the benefits of partial Fourier, parallel imaging, and CS strategies and thus provides the advantages of conjugated symmetry of k-space for multichannel parallel acquisition. Image compressibility facilitates complete use of signal redundancy and optimizes the scanning sequences. In this study, the feasibility of assessing left ventricular systolic function and 3D LV global and regional strain by using the uCS technique was investigated, and the agreement between CSC and CC protocols was determined.

## Materials and methods

### Patient population

A total of 37 consecutive volunteers were enrolled from February 2021 to April 2021. Of the total participants, seven volunteers who were unable to hold their breath for 30 s were excluded. Volunteers with cardiovascular diseases, diabetes mellitus, and systemic disorders were included. Finally, 30 volunteers were enrolled in this study. The study was conducted in accordance with the Declaration of Helsinki (as revised in 2013). The study was approved by the ethics committee of our institute, and written informed consent was obtained from each volunteer.

### Cardiac magnetic resonance protocol

Cardiac magnetic resonance images were acquired using the 1.436 T MRI system (uMR586, United Imaging, Shanghai, China) equipped with a maximum gradient field strength of 33 mT/m, a gradient slew rate of 125 T/m/s, and a 12-channel body phased-array coil. All volunteers were trained to hold their breath before the examination to ensure that they could hold their breath as long as possible during the examination. The training goal of BH was set above 30 s. All volunteers underwent a non-contrast CMR examination. CMR cine was performed using the balanced steady-state free precession sequence type. In the uMR586 MRI system, the k-space sampling model of uCS is specially designed for different scenarios. In dynamic imaging, randomized sampling is performed in the phase encoding direction (2D), as well as along the temporal dimension. Parallel acquisition is also performed along with randomized sampling. uCS reconstruction is performed by solving an optimization problem *via* the iterative method. The conventional technologies (partial Fourier, parallel imaging, and CS) are individual regularization items that are integrated by properly determined weightings. In the uCS reconstruction, the features of partial Fourier, parallel imaging, and compressed sensing are integrated and simultaneously contributed to the solution. Errors introduced by one technique will be suppressed or balanced by the others. Iterations continue until the reconstruction errors have reached an acceptable threshold. The CMR protocol included CC and CSC, with LV two-, three-, and four-chamber and SA views. The coverage of LV SA view cine was from apex to base, comprising a stack of nearly 8–12 slices. All CMR cines were performed with ECG gating and BH. CSC protocols were performed only on the SA view, following the CC protocol. CSC protocols included one BH CSC and the shortest BH CSC protocols with different parameters (Table 1). The scanning time of different SA cine sequences was recorded manually.

**TABLE 1:** Typical cardiovascular magnetic resonance (CMR) sequence parameters for conventional and compressed sensing short-axis cines.

Sequences parameters	CC	One BH CSC	Shortest BH CSC
ECG gating	Retrospective gating	Retrospective gating	Retrospective gating
uCS factor	–	3.0	3.0
FOV	(280–320) × (280–320)	(280–320) × (280–320)	(280–320) × (280–320)
Slice numbers	8–12	8–12	8–12
Slice thickness/gap(mm)	8/2	8/2	8/2
Flip angle(°)	70	70	70
TE/TR (ms)	1.58/3.36	1.58/3.36	1.58/3.36
Bandwidth(HZ/pixel)	1200	1200	1200
Image matrix(mm^2^)	192 × 100	192 × 100	192 × 100
Spatial resolution(mm^2^)	1.67 × 1.67	1.67 × 1.67	1.67 × 1.67
Cardiac phases	25	25	25
Views per segment	20	30–40	50–56
Breath holds(n)	7–11	1	1
Acquisition time(s)	72.97 ± 16.13(55.8–120.08)	20.84 ± 3.204(17–29.7)	14.22 ± 2.369(11–19.8)

CC, conventional cine; one BH CSC, one breath-hold compressed sensing cine; shortest BH CSC, shortest breath-hold compressed sensing cine; ECG, electrocardiograph; uCS, United Imaging compressed sensing; FOV, field of view; VPS, views per segment.

### Cardiac magnetic resonance analysis

The post-processing analysis of CMR images was performed using a dedicated software (CVI 42, version 5.13.5, Circle, Calgary, Canada). Imaging analysis included the following three components: visual assessment of SA cine image quality between protocols; LV volume and EF evaluations; and LV global and regional strain analyses.

### Image quality assessment

For the visual assessment of SA cine image quality, the image quality was graded on a 5-point scale (non-diagnostic = 1, poor = 2, adequate = 3, good = 4, and excellent = 5). An experienced radiologist with more than 10 years in CMR assessed the image quality, which included myocardial-blood contrast, endocardial border definition, and determining the presence of artifacts. Artifacts included those that were caused by respiration, mitigation, parallel imaging reconstruction, and residual motion, as well as CS-related staircase artifacts and radial streak artifacts (16, 18).

### Left ventricular volume evaluation

Short-axis view cines were subjected to post-processing for the evaluation of LV volume and EF. CC, one BH CSC, and shortest BH CSC images were analyzed sequentially every other month. LV end-diastole volume (EDV), end-systole volume (ESV), stroke volume (SV), and EF were calculated using the Simpson method in the function SAX module. The LV volume was covered from the apex to the annulus of the mitral valve and included papillary muscles. The endomyocardium was reviewed and corrected manually after LV automatic segmentation. For basal descent, slices were considered to be within the LV if the chamber was surrounded by at least 50% of the LV myocardium. The LV outflow tract was included in the LV blood volume. The endomyocardial contour was detected to include the LV outflow tract to aortic valve cusps on the basal slices (19).

### Strain analysis

**FIGURE 1:**
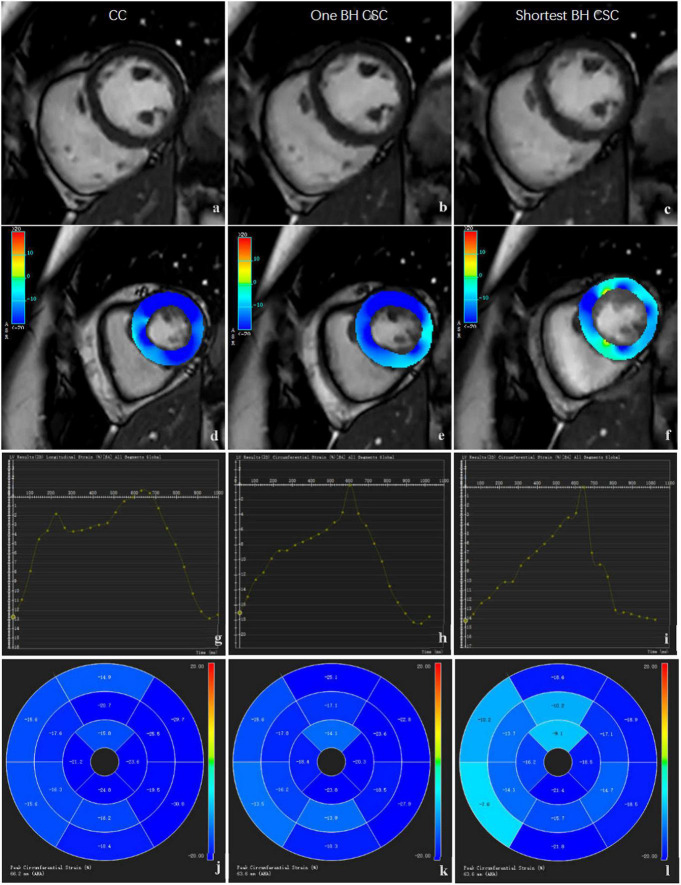
Comparison of SA images and strain analysis by different protocols. The images in left column **(a,d,g,j)**, middle column **(b,e,h,k)**, and right column **(c,f,i,l)** were acquired by CC protocols. The original SA images **(a,b,c)** are shown in the first row, strain color images **(d,e,f)** are shown in the second row, global strain curve **(g,h,i)** are shown in the third row, and regional strain polar maps **(j,k,l)** are shown in the fourth row.

For LV global and regional strain assessments, the strain module of CVI software was used to assess myocardial deformation. To perform LV 3D strain analysis, SA, two-, three-, and four-chamber view cines were added to this module. An auto contour detection using artificial intelligence was performed first, which was followed by a manual check. If the automatic segmentation of SA or long-axis view series was not satisfactory, the definitions of the mitral valve and apex, LV endo-, and epicardial contours at end-diastole phase on SA and long-axis cines were corrected. We then performed the strain analysis, and another adjustment was made if the strain borders were not satisfactory. Finally, 3D LV strain results were generated after the calculation was rerun (Figure 1). The LV global strain included GRS, GCS, and GLS. LV regional strain included basal, mid, and apical strains at each level. The strain consisted of regional radial strain (RRS), regional circumferential strain (RCS), and regional longitudinal strain (RLS). Similar to LV volume and EF evaluations, strain analysis for CC, one BH CSC, and shortest BH CSC images was performed sequentially every other month.

### Reproducibility

To test the reproducibility of strain analysis, CC images were used to assess intra- and inter-observer agreement in all volunteers. The strain analysis was performed separately by two observers with more than 8 years of CMR experience in post-processing. For inter-observer reliability, each observer performed the strain analysis, recorded the results, and reset the workspace, while ensuring that the first observer remained blinded to the results obtained by the second observer. One observer repeated the analysis after 1 month to determine the inter-observer agreement.

### Statistical analysis

The categorical data are presented as frequency and percentage. The quantitative data are presented as mean ± SD. The Shapiro–Wilk test was performed to assess the distribution of data. For normally distributed data, independent-sample Student’s *t*-test was performed for comparison. The Mann–Whitney U-test was performed for data with a non-normal distribution. To test the reproducibility of strain parameters, interclass correlation coefficient (ICC) was used for determining the inter- and intra-observer agreements. The Wilcoxon test was performed to compare image quality scores between different scan protocols. The Kendall correlation analysis was performed to test the agreement between the scores obtained by each protocol. For the agreement of LV volume, EF, global strain, and regional strain between different acquisitions, ICC, coefficient of variance (CV), and Bland–Altman plot were used. SPSS (version 20.0; IBM Corp., Armonk, NY, United States), MedCalc (version 18.2.1; MedCalc Software Ltd., Ostend, Belgium), and MS Excel (version 2019; Microsoft Corp., United States) were used for statistical analyses and plotting of graphs. All parameters with a *p* value of <0.05 were considered to be statistically significant.

## Results

### Baseline characteristics

Among the 30 enrolled volunteers, 10 were men and 20 were women. Notably, two volunteers had ventricular premature beat, one volunteer had breast cancer after chemotherapy and radiotherapy, and the remaining volunteers were healthy. The seven volunteers who were excluded had cardiovascular diseases, with three having coronary heart disease, two having cardiomyopathy, and two having heart failure. The age of the volunteers ranged from 21 to 60 years, with an average of 30.57 ± 11.26 years. The average heart rate was 73.67 ± 10.38 beats per minute, with a range from 59 to 92 beats per minute. The mean body mass index was 22.35 ± 3.95 kg/m^2^. The scanning time of SA cine using both CSC protocols was significantly shorter than that using the CC (72.97 ± 16.13 s) protocol. Moreover, the shortest BH CSC (14.22 ± 2.37 s) protocol was faster than one BH CSC (20.84 ± 3.20 s) protocol, and the difference was statistically significant.

### Image quality assessment

The scores of SA view cine image quality for CC, one BH CSC, and shortest BH CSC protocols were 4.73 ± 0.45, 4.67 ± 0.55, and 4.27 ± 0.58, respectively. The difference in image quality scores between the CC and one BH CSC protocols was not statistically significant (*p* = 0.16). However, the difference in scores between CC and shortest BH CSC protocols (*p* < 0.01) and between one BH CC and shortest BH CSC protocols was statistically significant (*p* < 0.01).

### Left ventricular systolic function

**FIGURE 2:**
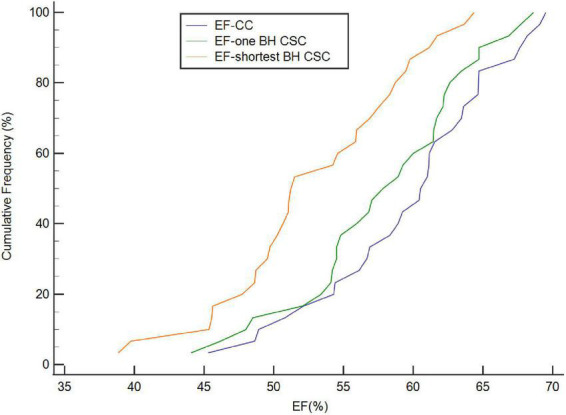
Cumulative frequency distribution of EF calculated by CC, one BH CSC, and shortest BH CSC acquisitions. EF calculated by both one BH CSC and shortest BH CSC acquisitions correlated well with that calculated by CC acquisition (ICC = 0.955 and 0.836, respectively).

Discrepancies in the LV volume and EF are shown between different acquisitions. Parameters such as EDV, SV, and EF were slightly lower and ESV was slightly higher with one BH CSC protocol compared with those obtained using the CC protocol; however, the differences were not statistically significant. The agreement of all variables between the CC and one BH CSC protocols was excellent (Figure 2). The differences between the CC and shortest BH CSC protocols were statistically significant, although the differences in SV and EF were not statistically significant (*p* = 0.03 and *p* < 0.01, respectively). Volumetric variables (EDV, ESV, and SV) between the CC and shortest BH CSC protocols also had a remarkable coherence (ICC > 0.9). The ICC of EF for the CC and shortest BH CSC protocols was also high (0.836), although it was lower than the ICCs for EDV, ESV, and SV. When CV was used to assess the agreement, CV results indicated a low variance between the CC and one BH CSC protocols. Most variables (EDV, ESV, and EF), except SV (CV = 15.57%), between the CC and shortest BH CSC protocols had a high but acceptable CV.

### Agreement of the global strain

**FIGURE 3:**
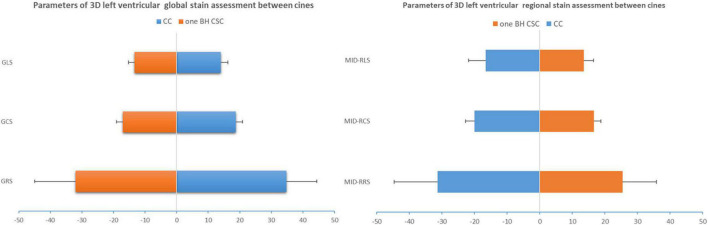
3D global strain and mid-regional strain computed by CC and one BH CSC protocols. ICCs of GRS, GCS, and GLS were greater than 0.8, and CVs of GCS and GLS were lower than 15%. ICCs were moderate (0.6 < ICC < 0.8) for mid-regional strain, but CVs were more than 15%.

**FIGURE 4:**
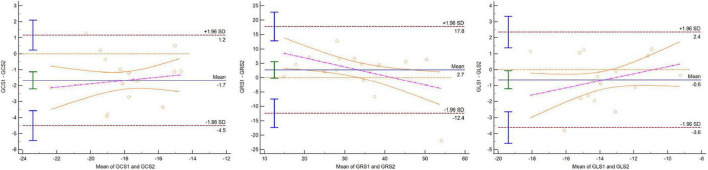
Bland–Altman plot indicating that 95% or more dots were located in 95% confidence interval for GCS, GRS, and GLS between CC and one BH CSC protocols.

The averages of GRS, GCS, and GLS were 37.72% ± 9.69%, -18.75% ± 2.18%, and -14.00% ± 2.28%, respectively, with the CC protocol. The global strain values by CSC sequences were lower than those by the CC protocol, as shown in Table 2 (*p* < 0.05). The values of GRS and GCS were higher with one BH CSC protocol (*p* > 0.05 for both) among the two CSC protocols (Figure 3). The agreement of 3D LV global strain assessment between the CC and one BH CSC protocols was good, as shown in Table 3. The Bland–Altman plot indicated that 95% or more dots were located in a 95% confidence interval (Figure 4). Between the CC and one BH CSC protocol, the ICCs of GRS, GCS, and GLS were greater than 0.8, and the CVs of GCS and GLS were lower than 15%. The ICCs of all variables were lower than 0.8, and the CVs of all parameters were higher than 15%, except for GLS, in both the CC and shortest BH CSC protocols. The differences in global strain were not statistically significant, except for GCS, between the protocols.

**TABLE 2:** 3D left ventricular global strain assessment between different acquisitions.

Variables	CC	One BH CSC	Shortest BH CSC	*p*-value1	*p*-value2
GRS(%)	37.72 ± 9.69	32.03 ± 12.92	28.89 ± 14.55	0.36	0.07
GCS(%)	-18.75 ± 2.18	−17.08 ± 1.99	−15.20 ± 1.72	<0.01	<0.01
GLS(%)	-14.00 ± 2.28	−13.37 ± 1.88	−13.38 ± 2.81	0.25	0.35

CC, conventional cine; one BH CSC, one breath-hold compressed sensing cine; shortest BH CSC, shortest breath-hold compressed sensing cine; ICC, intraclass correlation coefficient; CI, confidence interval; CV, coefficient of variance; GRS, global radial strain; GCS, global circumferential strain; GLS, global longitudinal strain; *p*-value1, CC group vs. one BH CSC group; *p*-value2, CC group vs. shortest BH CSC group.

**TABLE 3:** Agreement of 3D left ventricular global strain assessment between different acquisitions.

Variables	CC - one BH CSC				CC - shortest BH CSC			
		
	ICC	95%CI	CV (%)	95%CI (%)	ICC	95%CI	CV (%)	95%CI (%)
GRS	0.872	0.730–0.939	17.166	12.366–22.171	0.736	0.446–0.875	29.297	20.816–38.374
GCS	0.864	0.715–0.935	8.976	6.531–11.478	0.647	0.258–0.832	18.388	13.228–23.784
GLS	0.846	0.676–0.927	8.391	6.109–10.721	0.689	0.346–0.852	13.525	9.786–17.392

CC, conventional cine; one BH CSC, one breath-hold compressed sensing cine; shortest BH CSC, shortest breath-hold compressed sensing cine; ICC, intraclass correlation coefficient; CI, confidence interval; CV, coefficient of variance; GRS, global radial strain; GCS, global circumferential strain; GLS, global longitudinal strain.

### Agreement of the regional strain

**FIGURE 5:**
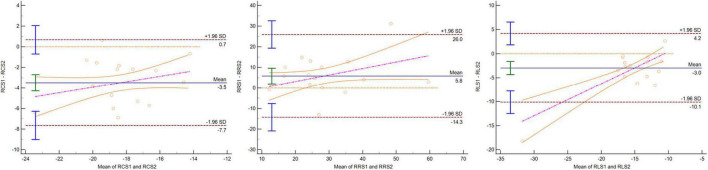
Bland–Altman plot indicating that 95% or more dots were located in 95% confidence interval for mid-RCS, mid-RRS, and mid-RLS between CC and one BH CSC protocols.

Three-dimensional LV regional strain assessment between the protocols showed an apparent discrepancy for basal, mid, and apical RRS (Table 4). The differences in apical-RCS and mid-RRS between one BH CSC and CC protocols were not statistically significant (*p* > 0.05). The regional strain values using CC acquisition were not always higher than those calculated by CSC acquisitions at the LV base and apex. The values of basal RRS and apical RLS with CC acquisitions were lower than those with one BH CSC and shortest BH CSC acquisitions, and the values with one BH CSC acquisition were greater than those with the shortest BH CSC acquisition. Unlike the global strain that showed good agreement between different protocols, the agreement of the regional strain between the CC and one BH CSC protocols was heterogeneous (Table 5). For the mid-regional strain and most basal regional strain variables, ICCs were moderate (0.6 < ICC < 0.8); ICCs of mid-regional strain variables were close to 0.8, and CVs were more than 15% (Figure 3). The agreement for regional strain variables at the base was mild, and CVs were also more than 15%, except for basal RCS. The ICCs of apical regional strain variables were mild or poor (-0.2 < ICC < 0.4) with heterogeneous CVs. The Bland–Altman plot indicated that 95% or more dots were located in a 95% confidence interval for mid-regional strain variables between the CC and one BH CSC protocols (Figure 5).

**TABLE 4:** 3D left ventricular regional strain assessment between cines.

Variables	CC	One BH CSC	Shortest BH CSC	*p*-value1	*p*-value2
**Basal**					
RRS(%)	27.98 ± 8.28	59.80 ± 21.10	47.60 ± 20.65	<0.01	<0.01
RCS(%)	−18.77 ± 2.66	−18.11 ± 1.88	−17.68 ± 1.93	<0.01	0.07
RLS(%)	−13.68 ± 2.81	−12.35 ± 2.84	−10.33 ± 7.46	0.03	0.01
Mid					
RRS(%)	33.32 ± 20.53	25.47 ± 10.27	22.75 ± 11.62	0.06	0.01
RCS(%)	−20.10 ± 2.73	−16.61 ± 2.19	−14.56 ± 2.05	<0.01	<0.01
RLS(%)	−16.55 ± 5.33	−13.59 ± 2.88	−12.95 ± 2.77	0.01	<0.01
Apical					
RRS(%)	59.80 ± 21.10	45.40 ± 77.98	30.62 ± 32.07	<0.01	<0.01
RCS(%)	−18.11 ± 1.88	−17.50 ± 3.31	−15.46 ± 2.63	0.39	<0.01
RLS(%)	−12.35 ± 2.84	−15.57 ± 6.54	−14.31 ± 3.16	<0.01	0.02

CC, conventional cine; one BH CSC, one breath-hold compressed sensing cine; shortest BH CSC, shortest breath-hold compressed sensing cine; RRS, regional radial strain; RCS, regional circumferential strain; RLS, regional longitudinal strain; *p*-value1, CC group vs. one BH CSC group; *p*-value2, CC group vs. shortest BH CSC group.

**TABLE 5:** Agreement of 3D left ventricular regional strain assessment between cines.

Variables	CC - one BH CSC			
	
	ICC	95%CI	CV (%)	95%CI (%)
**Basal**				
RRS	0.713	0.397–0.863	38.985	27.414–51.606
RCS	0.593	0.144–.806	14.632	10.573–18.841
RLS	0.691	0.350–0.853	22.862	16.361–29.726
Mid				
RRS	0.746	0.467–0.879	33.882	23.955–44.605
RCS	0.775	0.527–0.893	16.805	12.143–21.755
RLS	0.777	0.532–0.894	20.437	14.666–26.497
**Apical**				
RRS	-0.221	-1.565–0.419	122.054	79.879–174.118
RCS	0.435	-0.188–0.731	13.817	9.993–17.773
RLS	0.439	-0.178–0.733	30.390	21.567–39.853

CC, conventional cine; one BH CSC, one breath-hold compressed sensing cine; shortest BH CSC, shortest breath-hold compressed sensing cine; ICC, intraclass correlation coefficient; CI, confidence interval; CV, coefficient of variance; RRS, regional radial strain; RCS, regional circumferential strain; RLS, regional longitudinal strain.

### Reproducibility

The performance of CC images for strain analysis was excellent. Both intra- and inter-observer agreements were good. The ICCs of the intra-observer agreement for GRS, GCS, and GLS were 0.962, 0.963, and 0.938, respectively, whereas the ICCs of the inter-observer agreement for GRS, GCS, and GLS were 0.923, 0.924, and 0.931, respectively.

## Discussion

Compressed sensing cine sequence cardiovascular MRI could shorten the SA cine acquisition time to one or two BHs (10, 14, 15). The acquisitions in different studies have been shown to be inconsistent, and this applies not only to different CS acceleration factors but also to spatial, temporal resolution, flip angle, bandwidth, ECG gating, and cardiac phases. In our study, most of the 30 recruited volunteers were healthy, and BH for 30 s could be achieved after breath training. Therefore, we modified the one BH CSC protocol with the highest acceleration factor and increased views per segment to ensure that SA cine could be done in one BH. We only modified views per segment because we considered that the effect would be minimal if fewer parameters were changed, although the reduced temporal resolution was decreased. However, other protocols used a high CS factor and other parameters changed. One BH CS SA cine acquisition needs to be solved to keep all other parameters unchanged in the future.

Decreased scan time and the number of BHs are the advantages of CSC CMR (10, 15, 16). In our study, the duration of BHs for CC was longer than those for the one BH CSC and the shortest BH CSC protocols. SA cine acquisition includes a stack of 8–12 slices, and the CC protocol needs 7–11 intervals, which can extend the whole scan time to approximately 35–110 s, if each interval is set to 5 s. Long SA cine acquisition would increase the chance of arrhythmia occurrence or breathing motion during acquisition. Both the one BH CSC and the shortest BH CSC protocols involve one BH and markedly reduced scan time. For patients with premature beats, the CSC protocol is suitable for obtaining good image quality and EF analysis. Further studies (2, 20) have shown that the CSC protocol was feasible even in patients with tachycardia and atrial fibrillation, thus indicating a promising prospect for patients with arrhythmia. In this study, the duration of the BH CSC protocol was the shortest (11–19.8 s), which is still unsuitable for patients with arrythmia.

Image quality assessment indicated a slight difference between the one BH CSC and CC protocols. However, in the shortest BH CSC acquisition, more spatial and temporal blurring was observed by spatial domain pseudorandom sampling, especially in patients with a heart rate higher than 80 beats per minute. The main reason for the lower image quality could be inadequate views per segment. In overweight patients with a body mass index of >24 kg/m^2^, a few fold-over artifacts were found at the edges of images, although only a few involved the heart. Therefore, one BH CSC protocol with a lower view per segment would be a more feasible approach. A previous study (9) indicated that sensitivity-encoding sequences produced lower-quality diagnostic images and moderate artifact and noise than that produced by the gold standard 2D BH balanced steady-state free precession cine (8). CS could remove the artifacts in the image and improve the image quality as much as possible. The incoherent under-sampling acquisition makes artifacts irregularly scattered. Therefore, the consistency between the reconstructed image and the original image could be ensured using non-linear iterative reconstruction (21). In our study, the image quality score for one BH CSC protocol was slightly lower than that for the CC protocol, although the difference was not statistically significant. This result indicated an excellent consistency between the two sequences. This finding has been confirmed in other studies (10) using the signal-to-noise ratio to assess image quality.

Previous studies (9, 13, 14) on compressed sensing acquisition have reported an excellent agreement of the LV volume and EF value between the CSC and CC protocols. In our study, both the CSC protocols performed well in the LV volume and EF assessments. Specifically, one BH CSC protocol yielded an excellent agreement with CC acquisition, and the differences in all LV volumes and EF between the two acquisitions were small. This may improve examination efficiency and compliance of patients with mild disease capable of holding breath for >20 s. The agreement between the shortest BH CSC and CC protocols was also acceptable, with good ICC and CV values. The results indicated a potential value for patients with arrhythmia and reduced breath-holding performance. For patients with congenital heart disease, ischemic heart disease, cardiomyopathy, and heart failure, accurate LV volume and EF assessments are important for patient management. An alternative for patients who cannot hold breath for long could be the multiple BHs CSC protocol, in which the single BH time could also be decreased to an acceptable range. In an alternative CSC protocol, we could reduce the one BH duration to 3 s when breath holds were set at 8–12.

An incremental decrease in temporal under-sampling with increasing acceleration factors would lead to volumetric and functional parameter biases compared with fully sampled balanced steady-state free precession cine acquisition (22). Another study (23) indicated that BH CSC with high spatial resolution yielded a better image quality than that with CC acquisition; however, the LV volume and EF of BH CSC with high temporal resolution correlated well with those using CC acquisition. The parameter agreement was excellent between the one BH CSC and CC protocols, although we used a reduced temporal resolution. For the shortest BH CSC protocol, the image quality was reduced more for the worse temporal resolution, but the agreement was still acceptable. The underlying reason may be the fixed spatial resolution, bandwidth, and flip angle, which can affect both image quality and parameter agreement. Additionally, the majority of volunteers (24 volunteers) had a heart rate of <80 beats per minute, and the impact of reduced temporal resolution may be limited, especially in one BH CSC protocol. However, for volunteers who had a heart rate of >80 beats per minute using the shortest BH CSC protocol, the temporal resolution was too low to obtain good image quality, and this would also result in statistically significant biases. We believe that adequate temporal resolution is important for LV functional analysis, and reduced temporal resolution would also help shorten the scan time. Functional bias may also be related to different ECG gating protocols. Some studies (8, 24) have reported that CSC using prospective ECG triggering could be related to underestimated EDV and overestimated ESV. By contrast, another study (15) reported that adaptive ECG triggering and retrospective gating could provide the full cardiac cycle to overcome this limitation. Our study used retrospective gating for one BH CSC acquisition, and the acquired EDV and ESV were slightly different from those obtained with the CC protocol. Furthermore, we believe that retrospective gating could reduce the bias; however, the artifacts would also have affected the LV volume analysis. In addition, the heart rate may vary between the protocols, which is also related to volume biases.

For 3D global strain analysis, the performance of one BH CSC protocol was good but that of the shortest BH CSC protocol was not satisfactory. This indicates that the CC protocol may be replaced by one BH CSC protocol for 3D global strain analysis in some patients. A preliminary study (17) indicated that GCS was highly consistent between the CC and accelerated protocols (ICC = 0.884); however, the performance for GRS was unsatisfactory. The radial strain reflects wall thickness changes and is highly heterogeneous and sensitive to small differences. In another study (10) using the CS technique, the GCS obtained using the CSC and CC methods showed an excellent agreement (*r* = 0.95), and the mean difference was 0.1% with the limits of agreement between -2.8% and 3.0%. However, the global strain used in these studies was a 2D strain, which was calculated by SA view cine (10, 17). Additionally, GLS is an important index with a promising prognostic value in cardiovascular diseases (25–27). The findings of this study proved that not only GCS and GRS were consistent between one BH CSC and CC protocols but GLS was also potentially valuable. The global strain was calculated by short- and long-axis view cines and was a 3D global strain, with the whole left ventricle covered. The 3D strain included radial, circumferential, and longitudinal strains, whereas only radial and circumferential strains were acquired by 2D SA cine. The findings of our study further enhance the feasibility of the high-speed CSC technique in strain assessment.

Myocardial strain varies with changes in the spatial, temporal resolution, or cardiac phases. Variations in the spatial resolution may lead to GLS bias, as confirmed in a study on the effect of spatial resolution on myocardial strain using feature tracking (28). In recent studies (10, 13), the spatial resolution was reduced to acquire SA cine in one BH, and the global strain in these studies was variable because of differences in spatial resolutions. The low temporal resolution and decreased cardiac phases would also have affected the myocardial strain. For patients with a high heart rate, the reduced temporal resolution resulted in increased blurring, and this would have affected myocardial segmentation to make myocardial strain inconsistent. Similarly, decreased cardiac phases may miss the phases of the cardiac cycle, thus affecting the global strain and strain rates. Recent studies (10, 14, 15) have used fixed temporal resolution and alterable cardiac phases to assess the performance of the LV function, although the LV volume and EF using the CSC protocol were correlated with those obtained using the CC protocol. However, the cardiac phases were varying (19–31 phases), and the strain value may be non-negligible when the number of cardiac phases was <20 compared with the standard 25 cardiac phases. In addition, prospective ECG triggering could miss the very end of diastole, which may also cause a bias. In our study, we fixed the spatial resolution, cardiac phases, and ECG triggering; 3D global strain analyzed by one BH CSC protocol was consistent with the strain evaluated using the CC protocol, although the views per segment were increased. This finding would provide a practicable solution for global strain analysis using CSC acquisition. GLS has been used for evaluating prognosis in recent years (25–27). In this study, we found that GLS by one BH CSC protocol correlated with the index by the CC protocol. Of note, the CS technique was not used in the acquisition of long-axis view cines. We believe that the CS technique was meaningless in long-axis view cines because all these cines covered only one slice but not a stack of slices as SA cine and can be acquired in one BH using the CC protocol.

The regional strain analysis is also important in cardiac function assessment, especially in regional motion. Recent studies (29, 30) have indicated that regional circumferential and longitudinal strain impairment in patients with myocardial infarction could help in differentiating between damaged and normal myocardium. Another study (7) reported that subclinical LV dysfunction was correlated to the impaired regional strain for the lateral wall in myocarditis with a normal EF. In hypertrophic cardiomyopathy, the regional strain using speckle tracking echocardiography, CMR tagging, or feature tracking showed segmental dysfunction, and regional strain for hypertrophic segments or late gadolinium enhancement segments was worse (31–33).

To the best of our knowledge, this study is the first to assess the reliability of regional strain using the CS technique. Compared with the excellent reproducibility for the global strain analysis, the performance of regional strain analysis may not be robust enough. In healthy volunteers, the reproducibility showed that the ICCs for GRS, GCS, and GLS were excellent, but the apical-septal segment and basal anteroseptal segment showed increased CVs (4). In this study, the agreement of regional strain between one BH CSC and standard protocols was further explored, but the agreement assessment for regional strain between the shortest BH CSC and CC protocols was not assessed because the global strain agreement was not good enough. The findings showed a moderate-to-poor agreement between the protocols. Although the regional strain for the AHA 16 myocardial segments was not computed, the regional strain for apical, mid, and basal segments exhibited a varied consistency, whereas the regional strain for smaller segments exhibited a greater variation.

Most ICCs of <0.8 and CVs of >15% indicated that one BH CSC protocol cannot be used as an alternative to the CC protocol for regional strain analysis. However, the ICCs for mid-regional strain were close to 0.8, and CVs for mid-RCS and mid-RLS were close to 15%, indicating a promising efficacy of one BH CSC protocol. The agreement assessment results indicated worse regional strain for apical and basal segments. The underlying reason might be the variation of segmentation for basal and apical segments. At the basal level, the mitral annular increase and decrease would involve two or three SA slices, which may increase the difficulty of differentiation between the LV and atrial myocardium. At the apical level, the motion of the apex may also change the definition of apex at one or two SA slices. Moreover, the trabecular zone of the myocardium may affect the accuracy of endocardium and epimyocardium at the apex.

Another possible reason for the good performance of the regional strain analysis may be the image quality; additionally, reduced blood-myocardium contrast and focal artifacts may affect myocardial segmentation, spatial and temporal blurring, motion artifact, and interslice misalignment caused by the inconsistent breathing amplitude that may affect myocardial contouring and lead to inaccuracy of strain. In our study, both CS technique and increased views per segment could have contributed to the reduced image quality, but unfortunately, blood-myocardium contrast was not assessed. To date, one BH CSC has not been used for the feature tracking regional strain analysis. We believe that the regional strain analysis might be more sensitive to image consistency and details. Therefore, to improve the agreement of regional strain, further studies should focus on image quality improvement with adequate accelerated factors.

This study also has some limitations. First, we used a 1.436 T scanner whose signal-to-noise ratio is lower than that of 3.0T MRI, although artifact would be less. The second limitation is that most volunteers were healthy, some of whom could not hold their breath for 30 s, and two or three BHs CSC may be feasible in the clinical setting. The third limitation is that the image quality was graded visually, the blood-myocardium contrast ratio was not utilized, and the quantitative index was objective, indicating that the image quality assessment may not be accurate. Scanning time could be shortened with a decreased spatial flip angle and increased bandwidth, and acceleration could have been improved if these factors were involved in our study; however, these factors could affect the image quality. Therefore, further studies should focus on balancing image quality, functional parameter agreement, and acceleration.

The future of the CS technique is promising not only in highly accelerated SA cine acquisition but also in higher spatial and temporal resolutions that could improve the image quality (23). Moreover, the application in children and severely ill patients who cannot cooperate or hold breath well has been initially confirmed (13). Furthermore, the accelerated CS CMR protocol, especially in combination with the free-breathing protocol, may improve patients’ compliance (34, 35).

## Conclusion

The CS CMR technique may be useful in accelerated cine acquisition and cardiac volume and EF assessments. Our one BH CSC protocol further enhanced the clinical applicability of 3D global strain analysis; however, the one BH CS CMR protocol may still be unsuitable for regional strain assessment.

## Data availability statement

The raw data supporting the conclusions of this article will be made available by the authors, without undue reservation.

## Ethics statement

The studies involving human participants were reviewed and approved by the Affiliated Jinhua Hospital, Zhejiang University School of Medicine. Written informed consent to participate in this study was provided by the participants or their legal guardian/next of kin.

## Author contributions

HH: conception and design. JP: administrative support and provision of study materials or patients. YH and YP: collection and assembly of data. XC: data analysis and interpretation. All authors contributed to the manuscript writing and final approval of manuscript.
